# Left ventricular recovery in an African cohort of patients with peripartum cardiomyopathy

**DOI:** 10.11604/pamj.2024.47.6.42083

**Published:** 2024-01-05

**Authors:** Naïbé Dangwé Temoua, Joel Bamouni, Dakaboué Germain Mandi, Elisé Kaboré, Lucien Allawaye, Mianroh Hybi Langtar, Allamine Adjougoulta, Narcisse Douné, Ali Adam, Abdelmadjeib Zakaria, Yaméogo Rélwendé Aristide, Kambiré Yibar, Koudougou Jonas Kologo, Georges Rosario Christian Millogo, Nobila Valentin Yaméogo, Patrice Zabsonré

**Affiliations:** 1 Department of Cardiology, National Referral Teaching Hospital of N’Djamena, N’Djamena, Chad,; 2 Faculty of Human Health Sciences, University of N´Djamena, N´Djamena, Chad,; 3 Superior School of Health Sciences, University of Ouahigouya, Ouahigouya, Burkina Faso,; 4 Department of General Medicine, Cardiology Unit, Regional Hospital Center of Tenkodogo, Tenkodogo, Burkina Faso,; 5 Training and Research Unit of Health Sciences, University Professor Joseph Ki-Zerbo, Ouagadougou, Burkina Faso

**Keywords:** Peripartum cardiomyopathy, outcomes, recovery, Africa

## Abstract

Peripartum cardiomyopathy (PPCM) is a rare and potentially life-threatening disease associated with pregnancy. There are limited data regarding the outcome of PPCM and its predictive factors in sub-Saharan African patients. We prospectively conducted a double-center (cardiology unit of the department of medicine, Regional Hospital Center of Tenkodogo, Burkina Faso and the department of cardiology of the National Referral Teaching Hospital of N´Djamena, Chad) cohort study in patients with PPCM. Patients were consecutively enrolled from January 2015 to December 2017. Outcomes of interest were left ventricular recovery and poor outcome at one year. Ninety-four patients enrolled with a median age of 28 years. At one-year follow-up, 40.5% of them recovered their left ventricular function. Cox multiple regression analysis revealed that higher left ventricle ejection fraction (LVEF), lower natremia and use of betablockers were baseline variables predicting this end-point. Of the entire study population, 26.60% exhibited the composite end-point of death (n=15) or remaining in New York Heart Association (NYHA) class III-IV or LVEF < 35%. Predictors of poor outcome were lower LVEF at baseline, hyponatremia and use of digoxin. The current cohort study demonstrated that PPCM in sub-Saharan Africa is associated with limited myocardial recovery and significant rate of poor outcome at one year. Therefore, additional studies are needed to better address the disease.

## Introduction

Peripartum cardiomyopathy (PPCM) is an idiopathic form of cardiomyopathy presenting with heart failure secondary to left ventricular (LV) dysfunction towards the end of pregnancy or in the months following delivery, where no other cause of heart failure is identified [1]. It is an uncommon complication of pregnancy that remains a potentially severe maternal disease [1,2]. However, it has been shown that this condition is associated with a higher rate of spontaneous recovery of left ventricular function compared to other types of non-ischemic cardiomyopathy [3]. Worldwide studies reported marked heterogenicity in PPCM outcomes with many patients recovering their left ventricular function completely, however a considerable percentage remain in persistent dilated cardiomyopathy and chronic progressive heart failure [4-8]. Few data have assessed the long-term outcome of PPCM in sub-Saharan Africa. Therefore, we aimed to prospectively investigate the one-year left ventricular recovery and its predictive factors in patients with PPCM from Burkina Faso and Chad.

## Methods

**Study settings:** this prospective double-center cohort study was conducted at both cardiology units of the department of medicine, Regional Hospital Center (RHC) of Tenkodogo, Burkina Faso (center 1) and the department of cardiology of the National Referral Teaching Hospital of N´Djamena (NRTHN), Chad (center 2). The RHC of Tenkodogo is the unique tertiary health care center that covers a dry orchard savannah region populated of about 1.4 million inhabitants, almost constituted by subsistence farmers. Patients were referred from regional primary and secondary public health care services, local private clinics and the Department of obstetrics of the Regional Hospital of Tenkodogo. The department of cardiology of the NRTHN, is the main of two cardiology units for the whole of Chad, all located in N´Djamena and receiving patients from countrywide.

**Study process:** from January 2015 to December 2016, women with diagnosed PPCM were consecutively recruited at two centers. Inclusion criteria were age ≥ 15 years; symptoms of congestive heart failure developed in the last month of pregnancy or during the last five months postpartum; no other identifiable cause of heart failure, sinus rhythm on a twelve leads electrocardiogram and LVEF ≤ 45% by transthoracic echocardiography. At the time of enrollment, each patient underwent a clinical examination, recording signs and symptoms and demographic characteristics. Blood analysis was also performed at baseline.

**Echocardiography assessment:** echocardiography was performed in all patients at study entry according to the American Society of Echocardiography (ASE) guidelines [9]. Left ventricular ejection fraction was assessed using Simpson´s biplane method and left ventricular end-diastolic diameter measured in the parasternal long-axis view and indexed with the body surface area. Left ventricle ejection fraction measurement was repeated at six months and one year. Medications received by patients were recorded. Furosemide was prescribed in all patients and titrated until congestive symptoms' relief. Considering the benefit of breastfeeding and its advantages over artificial feeding (in terms of costs, immunological and nutritional aspects, hygienic preparation conditions), infant survival in low-income regions [10], the prescription of bromocriptine was left optional depending on its availability and affordability at each center.

**Follow-up:** each patient was followed up for a one-year period, with NYHA functional class and LVEF being assessed at six- and twelve-months period.

**Outcomes of interest:** outcomes measured were left ventricular function recovery defined as a LVEF ≥ 50% and poor outcome defined as a composite endpoint of death or LVEF < 35% or remaining in NYHA functional class III-IV at one year.

**Statistical analysis:** data were analyzed using R Studio software version 1.2.5033. Continuous variables were expressed as median (IQR) and categorical variables as percentage. Differences between centers were assessed using Wilcoxon rank-sum test, chi-square test of independence or Fisher's exact test as appropriate. Kaplan-Meier method was used to construct the global survival curve. Cox univariate and multiple regression were performed to establish independent predictors of LV recovery, death and poor outcome. Backward elimination procedure according to Akaike´s Information Criterion (AIC) was used to remove the potential confounding variables and to determine in fine the best model. The proportional-hazards assumption was done with the Schoenfeld individual Test to validate the final Cox model. A two-sided p value of < 0.05 was considered statistically significant.

**Ethic aspects:** the study was approved by the institutional review boards at participating centers. The board waived the need for signed written informed consent due to illiteracy of most study participants and that collected data were non-invasive and derived from routine practice. Only oral informed consent was required. The study was carried out in accordance with the principles of the Declaration of Helsinki [11].

## Results

**Figure 1:**
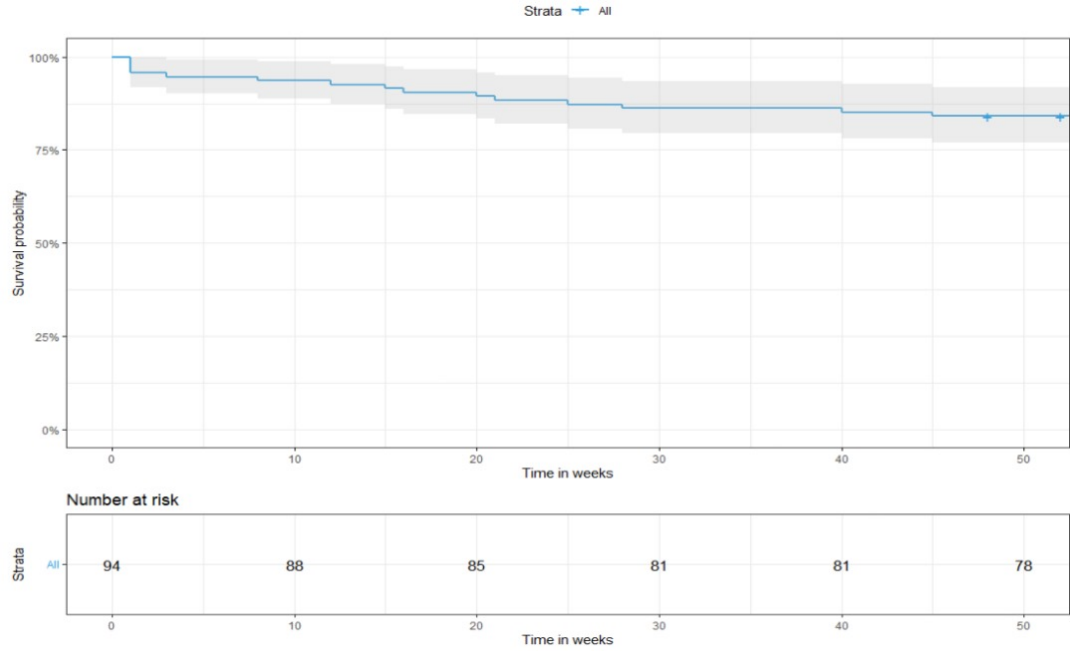
Kaplan-Meier survival curve in patients with Peripartum cardiomyopathy

Overall, 94 patients were enrolled into the study (34% from Burkina Faso and 66% from Chad), with a median age of 28 years (IQR: 21, 32), parity of 4 (IQR: 2.0, 6.0), gravidity of 3 (IQR: 1.0, 6.0). The median length of symptoms onset at study entry was 45 days (IQR: 21, 84) and most of the women (95%) presented with NYHA functional class III-IV symptoms. The median LVEF at baseline was 31.5 % (IQR: 28.0, 36.0) and LVEDD index was 38.5 mm (IQR: 34.9, 40.6). Patients were given beta blockers (48%), angiotensin-converting enzyme inhibitors or angiotensin receptor blockers (98%) and spironolactone (95%). Forty-one women were treated with bromocriptine. Centers were similar when comparing baseline characteristics apart from bromocriptine that was only prescribed in center 2, digoxin and natremia (p < 0.05) (Table 1). For the entire cohort, the median LVEF at 6 months and 12 months was 40 % (IQR: 28,36) and 45 % (IQR: 34,58), respectively. Follow-up median LVEFs did not differ by center at 6 months (Burkina Faso=44.6 (IQR: 31, 52.5), Chad= 39 (IQR: 30, 55); p-value = 0.69) and 12 months (Burkina Faso= 50 (IQR: 34, 58), Chad= 39 (IQR: 32, 58); p-value = 0.16). Women with LVEF < 30% at time of enrollment (n=32), exhibited a lower median LVEF at 6 months (28 (IQR: 22, 39) versus 46.8 (IQR: 35, 57); p < 0.001) and at 12 months (IQR: 32 (27, 41) versus 51 (IQR: 38, 60); p < 0.001). At one-year follow-up, 32 (40.5%) of the 79 surviving women, had fulfilled the LV recovery end-point. Twenty-six (33%) of them partially recovered their LV function (LEVF between 35% and 49%). Cox univariate analysis demonstrated that length of symptoms at study entry, LVEF, natremia and use of beta blockers were associated with LV recovery. Stepwise multiple regression analysis showed that higher baseline LVEF, natremia, and use of beta blockers were predictors of 12 months LV recovery (Table 2). During the 12-month follow-up period, of the entire study population, 25 (26.60%) met the composite end-point of death (n=15) or remaining in NYHA class III-IV or LVEF < 35%. Both univariate and stepwise multiple regression analysis demonstrated that predictors of poor outcome were lower LVEF at baseline, hyponatremia and use of digoxin (Table 3). The overall survival rates were 92.6% at 3-month, 87.6% at 6-month and 84% at 12-month follow-up as shown in Figure 1.

**Table 1:** baseline characteristics of study patients and outcome over time

Parameter	All (N = 94)	Center 1 (n = 32)	Center 2 (n = 62)	P value
Age (years)	28 (21, 32)	30 (24, 32)	26 (20, 33)	0.089
Parity	4.0 (2.0, 6.0)	3.0 (2.0, 6.0)	4.0 (2.0, 6.0)	0.6
Gravidity	3.0 (1.0, 6.0)	3.0 (2.0, 5.0)	3.5 (1.0, 6.0)	0.6
BMI (kg/m^2^)	20.1 (18.2, 22.3)	20.5 (18.7, 22.4)	19.7 (17.6, 22.0)	0.2
Length of symptoms at presentation (days)	45 (21, 84)	35 (21, 63)	56 (16, 84)	0,7
SBP, mmHg	120 (100, 130)	115 (100, 120)	120 (110, 130)	0.2
DBP, mmHg	80 (70, 80)	75 (70, 80)	80 (70, 80)	0.2
Heart rate (beats/min)	110 (100, 120)	108 (94, 120)	110 (100, 120)	0.11
NYHA III-IV	89 (95%)	27 (84%)	62 (100%)	NA
Hemoglobin (g/dl)	11.5 (10.3, 12.6)	11.8 (10.8, 12.5)	11.4 (9.9, 12.7)	0.2
Natremia (mEq/l)	138 (136, 140)	139 (137, 141.2)	137.5 (135, 139)	0.002
eGFR (ml/min/1.73m^2^)	94 (84, 100)	88 (76, 99)	95 (87, 101)	0.054
LVEF	31.5 (28.0, 36.0)	31.3 (28.0, 37.0)	31.5 (27.2, 35.8)	0.7
LVEDD index	38.5 (34.9, 40.6)	37.1 (34.8, 40.3)	39.1 (35.7, 40.7)	0.7
TAPSE, mm	16.0 (13.0, 18.0)	16.0 (13.0, 18.0)	17.0 (13.0, 18.0)	0.4
ACEi/ARB	92 (98%)	32 (100%)	60 (97%)	NA
Betablockers	45 (48%)	15 (47%)	30 (48%)	>0.9
Anti-aldosterone	89 (95%)	32 (100%)	57 (92%)	NA
Digoxin	21 (22%)	12 (38%)	9 (15%)	0.023
Bromocriptine	41 (44%)	0 (0%)	41 (66%)	NA
LVEF at 6 months	42 (32, 55)	47 (39, 55)	39 (30, 55)	0.2
LVEF at 12 months	45 (34, 58)	50 (36, 58)	39 (33, 58)	0.2
Recovery at 6 months	26 (28%)	10 (31%)	16 (26%)	0.8
Recovery at 12 months	32 (34%)	16 (50%)	16 (26%)	0.034
One-year poor outcome	38 (40%)	10 (31%)	28 (45%)	0.3
One-year death	15 (16%)	2 (6.2%)	13 (21%)	0.12

IQR: interquartile range; BMI: body mass index; SBP: systolic blood pressure, DBP: diastolic blood pressure; NYHA: New York Heart Association; NA: not applicable; LVEF: left ventricular ejection fraction; LVEDD: left ventricular end-diastolic diameter; TAPSE: tricuspid annulus plane systolic excursion; eGFR: estimated glomerular filtration rate using CKD-EPI equation; ACEi: angiotensin-converting enzyme inhibitors; ARB: angiotensin receptor blockers

**Table 2:** Cox univariate and multivariate regression analysis predictors of one-year LV recovery (n=79)

Characteristic	Univariate			Multivariate (AIC = 79.73)		
	OR	95% CI	p-value	OR	95% CI	p-value
Age	1.01	0.83 - 1.24	>0.9			
Parity	2.68	0.63 - 13.4	0.2	2.70	0.70 - 12.2	0.2
Gravidity	0.25	0.04 - 1.15	0.10	0.23	0.04 - 0.98	0.063
BMI	0.87	0.66 - 1.10	0.3			
Length of symptoms	0.93	0.86 - 0.99	0.04	0.94	0.87 - 0.99	0.06
SBP	1.04	0.97 - 1.11	0.3	1.04	1.00 - 1.09	0.10
NYHA class III-IV	0.05	0.00 - 23.5	0.6			
Hemoglobin	1.53	0.87 - 2.91	0.2	1.58	0.99 - 2.73	0.073
Natremia	1.33	1.07 - 1.75	0.018	1.33	1.11 - 1.64	0.004
eGFR	1.01	0.96 - 1.06	0.8			
LVEF	1.26	1.06 - 1.59	0.021	1.31	1.13 - 1.60	0.002
LVEDD index	0.86	0.65 - 1.05	0.2			
TAPSE	1.12	0.88 - 1.45	0.4			
Betablockers	5.86	2.33 - 16.05	0.0002	4.32	1.14 - 18.9	0.037
Digoxin	0.42	0.03 - 4.81	0.5			
Bromocriptine	0.51	0.08 - 2.77	0.4			

AIC: Akaike information criterion; OR: Odds Ratio; CI: confidence interval; BMI: body mass index; SBP: systolic blood pressure; NYHA: New York Heart Association; LVEF: left ventricular ejection fraction; LVEDD: left ventricular end-diastolic diameter; TAPSE: tricuspid annulus plane systolic excursion; eGFR: estimated glomerular filtration rate (ml/min/1.73 m^2^) using CKD-EPI equation

**Table 3:** Cox univariate and multivariate regression analysis predictors of one-year poor outcome (n=94)

Characteristic	Univariate			Multivariate (AIC=89.15)		
	OR	95% CI	p-value	OR	95% CI	P-value
Age	0.95	0.79 - 1.12	0.6			
Parity	2.05	0.80 - 5.39	0.13			
Gravidity	0.57	0.22 -1.39	0.2			
BMI	0.88	0.68 -1.11	0.3			
Length of symptoms	1.02	0.97 - 1.07	0.4			
SBP	1.04	0.99 - 1.11	0.15			
NYHA class III-IV	1.26	0.01 – 22	>0.9			
Hemoglobin	1.33	0.83- 2.23	0.2			
Natremia	0.87	0.70 -0.96	0.009	0.87	0.75 -0.99	0.048
eGFR	1.004	0.98 -1.03	0.95			
LVEF	0.85	0.78 - 0.93	<0.001	0.82	0.72 -0.91	<0.001
LVEDD index	0.97	0.82 -1.14	0.7			
TAPSE	0.97	0.79 - 1.17	0.7			
Betablockers	0.20	0.03, 0.91	0.050			
Digoxin	7.41	2.56 - 25.04	<0.001	5.86	1.53 - 26.0	0.013
Bromocriptine	2.19	0.50 -11.2	0.3			

AIC: Akaike information criterion; OR: Odds Ratio; CI: confidence interval; BMI: body mass index; SBP: systolic blood pressure; NYHA: New York Heart Association; LVEF: left ventricular ejection fraction; LVEDD: left ventricular end-diastolic diameter; TAPSE: tricuspid annulus plane systolic excursion; eGFR: estimated glomerular filtration rate (ml/min/1,73 m^2^) using CKD-EPI equation

## Discussion

This prospective cohort study has shown the prognosis of PPCM patients who benefited from standard heart failure medical treatment in a sub-Saharan African context. At one-year follow up, only 34% of patients recovered their left ventricular function and 40% exhibited a poor outcome. These findings are consistent with data from most African series [12-14]. In contrast, data from developed countries reported better outcome. Indeed, McNamara *et al*. [15] have found a left ventricular recovery rate of 72% with only 13% of their study patients having poor outcome. Poorer outcome may be in part due to initial worse clinical pattern of heart failure at presentation; socio-economic inequities; low access to good quality of care, particularly for PPCM patients from low-income settings. It has been shown that delays in diagnosis may result in lower LVEF at diagnosis and subsequent lower recovery rates [16]. Baseline LVEF < 30% and a LVEDD > 60mm are shown to be strongly associated with lower LVEF recovery at one year [16,17]. Delays in diagnosis contribute to retardation of heart failure treatment initiation, favoring myocardial fibrosis and then may lead to the onset of irreversible cardiomyopathy similar to the natural history of idiopathic cardiomyopathy. Therefore, improving heart failure patients´ care pathway is useful in terms of PPCM prognosis [18]. As shown in the present paper, the mortality rate in developing countries was significantly higher than that in advanced countries (14%, 95% CI: 10 - 18% versus 4%, 95% CI: 2 - 7%, p< 0.001) [19]. Lower socio-economic conditions and lack of health insurance are partly involved in jeopardizing early initiation and continuation of treatment in patients with chronic disease. Moreover, repeated treatment discontinuation as previously shown in our setting [20] may contribute to PPCM poor outcome. Our study may be limited by the lack of some socio-demographic parameters (such as socio-economic condition, educational level, marital status geographic access to health facilities...) during data acquisition and analysis process.

## Conclusion

This prospective cohort study revealed that less than a half of sub-Saharan African women with PPCM recovered myocardial function at one-year follow-up. Moreover, a significant number died or developed persistent severe cardiomyopathy. Further studies are needed to better understand and address the underlying causes of such huge burden of PPCM.

### What is known about this topic

- *Peripartum cardiomyopathy is an uncommon complication of pregnancy that remains a potentially severe maternal disease;*
- *Compared to other types of non-ischemic cardiomyopathy, Peripartum cardiomyopathy is known to be associated with higher rate of myocardial recovery*.

### What this study adds

- *Peripartum cardiomyopathy in sub-Saharan exhibited poorer outcome when compared with western regions;*
- *Conventional therapy should be made available and affordable for Peripartum cardiomyopathy patients in order to increase recovery rates*.
